# Spectroscopic Characterization of Natural Melanin from a *Streptomyces cyaneofuscatus* Strain and Comparison with Melanin Enzymatically Synthesized by Tyrosinase and Laccase

**DOI:** 10.3390/molecules23081916

**Published:** 2018-08-01

**Authors:** Maher Al Khatib, Mohamed Harir, Jessica Costa, Maria Camilla Baratto, Irene Schiavo, Lorenza Trabalzini, Simona Pollini, Gian Maria Rossolini, Riccardo Basosi, Rebecca Pogni

**Affiliations:** 1 Department of Biotechnology, Chemistry and Pharmacy, University of Siena, 53100 Siena, Italy; maher.alkhatib@student.unisi.it (M.A.K.); jessica.costa@student.unisi.it (J.C.); mariacamilla.baratto@unisi.it (M.C.B.); schiavo6@student.unisi.it (I.S.); lorenza.trabalzini@unisi.it (L.T.); riccardo.basosi@unisi.it (R.B.); 2 CSGI (Consorzio per lo Sviluppo dei Sistemi a Grande Interfase), 50019 Florence, Italy; 3 Biology of Microorganisms and Biotechnology Laboratory, University of Oran 1 Ahmed ben Bella, BP1524, Oran El Mnaouer, 31000 Oran, Algeria; mohamedharir57@yahoo.fr; 4 Algeria and Faculty of Sciences, Natural and Life Sciences Department, Mohamed Boudiaf University, M’sila, Algeria; 5 Department of Experimental and Clinical Medicine, University of Florence and Clinical Microbiology and Virology Unit, Careggi University Hospital, 50134 Florence, Italy; simona.pollini@unifi.it (S.P.); gianmaria.rossolini@unifi.it (G.M.R.)

**Keywords:** *S. cyaneofuscatus* Pridham et al. 1958 melanin, melanogenesis enzymes, melanin spectral characterization, radical species, multifrequency electron paramagnetic resonance (EPR)

## Abstract

An actinobacteria strain was isolated from Algerian Sahara soil and assigned to *Streptomyces cyaneofuscatus* Pridham et al. 1958 species. This strain was selected for its ability to produce melanin exopigments in liquid and solid media. Melanin synthesis was associated with tyrosinase activity and the enzyme from this strain was isolated and biochemically characterized. Synthetic melanin was then enzymatically produced using the *S. cyaneofuscatus* Pridham et al. 1958 tyrosinase. As this enzyme showed a higher diphenolase activity, a synthetic melanin from the enzymic oxidation of 3,4-dihydroxyphenylalanine (dopa) was obtained by the use of a *Trametes versicolor* (L.) Lloyd laccase for comparison. The natural and synthetic pigments were physico-chemically characterized by the use of ultraviolet (UV)-Visible, and Fourier transform infrared (FT-IR) and multifrequency electron paramagnetic resonance (EPR) spectroscopies. All the melanin samples displayed a stable free radical when analyzed by X-band EPR spectroscopy. Once the samples were recorded at Q-band EPR, a copolymer derived from a mixture of different constituents was evident in the natural melanin. All radical species were analyzed and discussed. The use of water-soluble melanin naturally produced by *S. cyaneofuscatus* Pridham et al. 1958 represents a new biotechnological alternative to commercial insoluble pigments.

## 1. Introduction

Melanins are pigments exhibiting unique properties as ultraviolet (UV)-visible light absorbers, free radical scavengers, and metal ions chelators [1,2,3,4,5]. Their molecular structure has not been univocally defined, depending on the polymerization conditions [6,7,8]. Computational models have been successfully used to investigate eumelanins as polymers that have a high level of chemical and geometrical disorder, so that the term polymer can be used to refer to their polymer-like properties [9,10]. In particular, their monotonously increasing light absorption from the visible to ultraviolet region has supported this description [9]. Melanins are promising pigmented conductive coatings for material technology and in the field of optoelectronics, as a large proportion of the new melanin applications rely on their efficient photon-phonon coupling [3,9,11]. The combination of these properties, together with a better understanding of salient features at the structural level, could dramatically increase the range of applications of this polymer in the field of bio-electrochemistry or energetics. Structural tuning could be used to produce melanins mimicking properties characteristic of other natural relevant molecules, such as the strong adhesive properties of mussel proteins [1]. 

Melanins are brown to black pigments found in animals, plants, and microorganisms. Melanins are formed from phenolic compounds by polymerization via quinones, whose formation is catalyzed by phenoloxidases. Many organisms, including plants and fungi, use phenols lacking nitrogen (N) by N economy. In animals, black eumelanin and reddish-yellow pheomelanin are produced by the enzymatic oxidation of tyrosine via l-3,4-dihydroxyphenylalanine (l-dopa) to dopa-quinone. The oxidative reaction involves enzymes like tyrosinase, laccase, or tyrosine hydroxylase, depending on the organism [12,13,14,15]. Subsequently, non-enzymatic reactions lead to the formation of the polymer-like structure. Eumelanins are generally defined as heterogeneous macromolecules characterized by the presence of different amounts of 5,6-dihydroxyindole (DHI) and its 2-carboxylated form (5,6-dihydroxyindole-2-carboxylic acid, DHICA). Pheomelanins differ from eumelanins by the presence of sulphur-containing cysteinyldopa (cd), which is directly formed by the cysteinylation of dopa or by the mediation of glutathione [2,16,17]. In fungi and other microorganisms, melanins are classified on the basis of their precursor molecules: 3,4-dihydroxyphenlyalanine (dopa), dihydroxynaphatalene (DHN), and homogentisic acid (pyomelanins). This group of polymers is classified as allomelanins, and differently from pheomelanins, they contain neither sulphur nor nitrogen [17,18]. Some species of bacteria and fungi secrete pyomelanins, produced by a different phenylalanine and tyrosine degradation pathway. This soluble melanin was identified as the main component in the *Sporothrix schenckii* and *Aspergillus fumigatus* pathogenic fungi [19,20].

Although both eumelanins and pheomelanins are well represented among mammals, few studies regarding the production of pheomelanins in bacteria have been published [21]. Pheomelanin was identified as the main component in the extracellular melanin produced by a marine strain of *Streptomyces* sp., in *Lachnum* YM404, in the fungus *Auricularia auricula*, and in the soil fungi *Cladosporium cladosporioides* [22,23,24,25].

An actinobacteria isolated from Sahara soil was molecularly characterized and assigned to *Streptomyces cyaneofuscatus* [13]. This microorganism is able to produce a considerable amount of water soluble melanin exopigments when it is grown on tyrosine containing agar media. A tyrosinase was isolated as the enzyme responsible for melanin formation [13]. Tyrosinases are bifunctional enzymes catalyzing two different reactions: the hydroxylation of monophenols to *o*-diphenols (cresolase activity) followed by the oxidation of *o*-diphenols to *o*-quinones (cathecolase activity), with the reduction of molecular oxygen to water. In general, tyrosinases from different *Streptomyces* species are characterized by a more pronounced diphenolase activity [26,27]. 

In this paper, the natural melanin was analyzed and compared with the enzymic melanin-like pigment synthesized by the *Streptomyces cyaneofuscatus* Pridham et al. 1958 tyrosinase and the one obtained by *Trametes versicolor* (L.) Lloyd (hereinafter referred to as “Tv”) laccase using dopa as the substrate. All the samples were characterized by the use of ultraviolet-visible (UV-Vis), Fourier transform infrared (FT-IR) and multifrequency electron paramagnetic resonance (EPR), also called electron spin resonance (ESR), spectroscopies. The EPR is considered the elective technique for identifying melanin samples by the permanent single slightly asymmetric signal recorded at X-band (microwave frequency approximately 9 GHz), at which the standard EPR spectrometers operate [2,16,28,29]. Nevertheless only the use of a continuous wave (cw) multifrequency EPR approach enables a sensitive and nondestructive analysis and differentiation of the natural melanin constituents when the complex copolymer eumelanin/pheomelanin is present [30,31,32,33]. In this context, the EPR spectra recorded at Q-band (microwave frequency approximately 35 GHz) and at S-band (microwave frequency approximately 4 GHz) were useful for the precise g-value determination (S-band) and to identify the presence of pheomelanin in the water-soluble *S. cyaneofuscatus* Pridham et al. 1958 melanin pigment (Q-band). The EPR spectra of natural melanin were compared with the enzymatically synthesized eumelanin and cysteinyldopa pigments to determine its composite nature. 

## 2. Results and Discussion

A strain belonging to the *Streptomyces cyaneofuscatus* Pridham et al. 1958 species, hereinafter referred to as “Sc-Ms1” [13], was isolated from Algerian Sahara soil and analyzed for its ability to produce melanin in solid and liquid media given the activity of an extracellular tyrosinase. This enzyme was then isolated and characterized [13]. 

**Figure 1:**
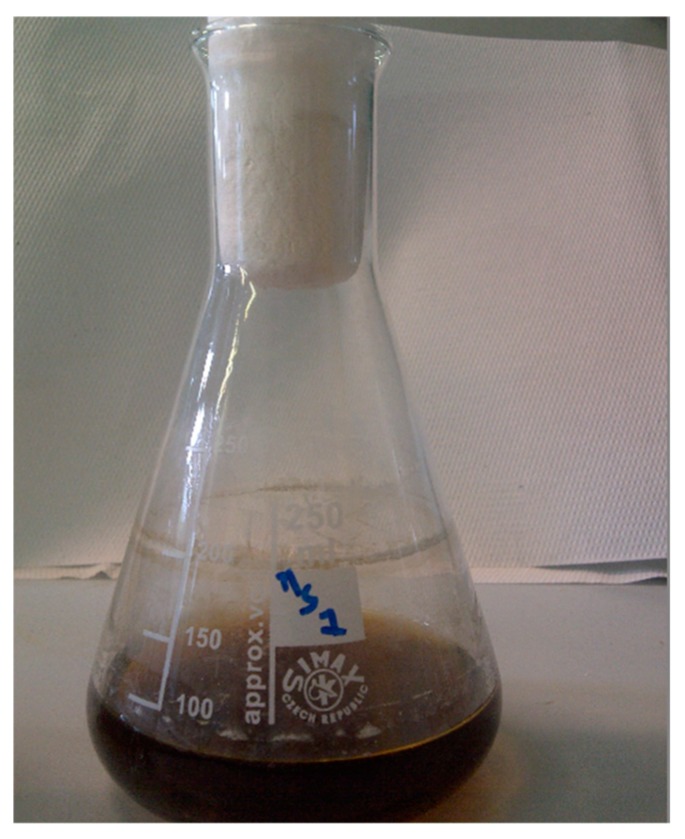
Melanin pigment of Sc-Ms1 in Modified Phenoxazinone Production Medium (MPPM) culture broth.

Figure 1 shows the liquid culture of the strain with the blackish-brown pigment diffused in the medium. 

The extracellular enzyme tyrosinase was partially purified by ammonium sulphate precipitation. The substrate specificity was tested toward different mono-, di-, and tri-phenols [13].

The Sc-Ms1 tyrosinase showed a higher substrate specificity for diphenols than monophenols. The low monophenolase/diphenolase activity is a common feature shared with other plant and bacterial tyrosinases [27,34,35]. The involvement of a laccase—a structurally different polyphenol oxidase—in the formation of water soluble melanin pigments in the *Bacillus weihenstephanesis* isolates was established [12]. For this reason, the enzymic melanin was described in this study referring to the melanin like-pigments synthesized by Sc-Ms1 tyrosinase and Tv laccase using the same experimental conditions [6].

**Figure 2:**
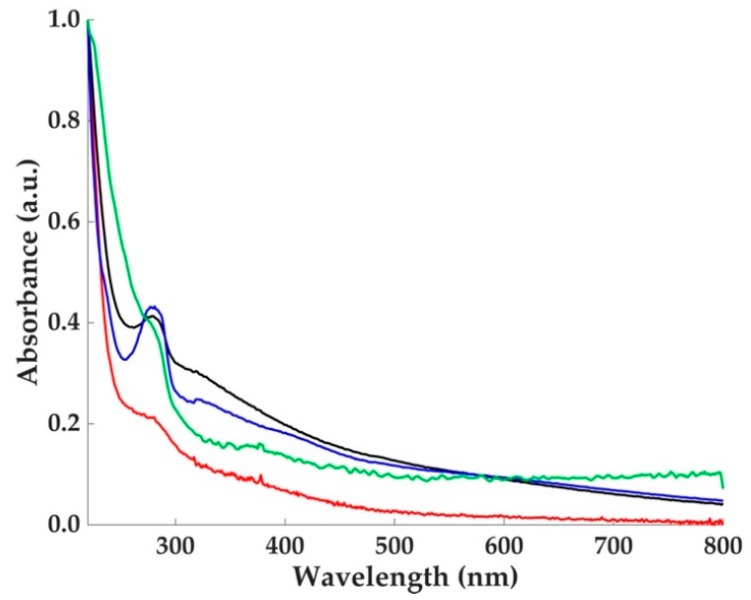
Ultraviolet-visible (UV-Vis) absorption spectra of Sc-Ms1 melanin (red line), melanin-like pigment synthesized by Sc-Ms1 tyrosinase (black line), dopa (blue line), and cysteinyldopa (green line) melanin-like pigments synthesized by Tv laccase. An excess of dopa was used as substrate for the enzymic synthesis. The peak at 274 nm is dependent on unreacted substrate and the presence of protein.

The UV-Vis spectra of the samples displayed a monotone increase in radiation absorption at lower wavelengths (Figure 2), the pigment providing an efficient photoprotective function. This behavior is typical and one of the most important features of melanins [2]. From the inspection of the absorption profiles reported in Figure 2, the spectrum of the water-soluble melanin (Figure 2, red line) had no detectable differences compared to the dispersed solution of dopa-melanin tyrosinase and dopa and cysteinyldopa laccase (Figure 2, black, blue, and green lines respectively). Furthermore, the region around 500 nm is featureless in all the spectra. The monotonic increase absorbance for the poorly solubilized eumelanin was ascribed to scattering effects, which are less effective in soluble natural melanins [2]. Regardless, the origin of the broadband absorption spectrum of melanins paired with the determination of their chemical structure have been the object of scientific debate. A model based on an interplay of geometrical order and disorder of eumelanin aggregate structures has been successfully used to describe the absorption spectra by a first-principles computational investigation [9]. 

**Figure 3:**
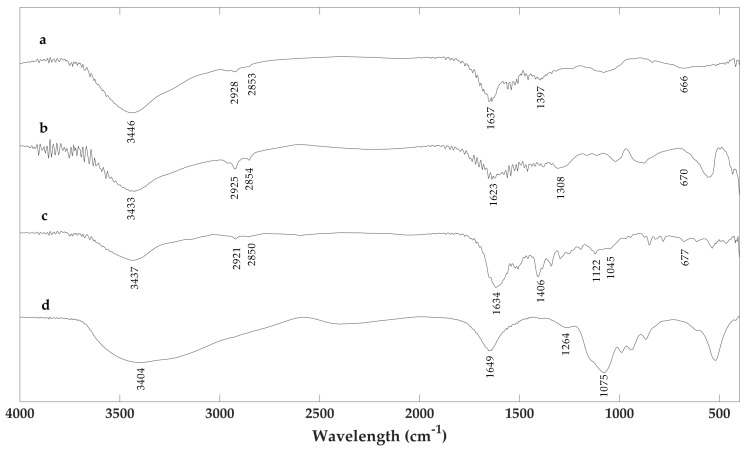
Fourier-transform infrared (FTIR) spectra of (**a**) Sc-Ms1 melanin, (**b**) Sc-Ms1 tyrosinase dopa-melanin, (**c**) Tv laccase cysteinyldopa melanin, and (**d**) Tv laccase dopa-melanin. All spectra were recorded in transmittance mode.

In Figure 3, the FT-IR spectra are reported. All spectra were characterized by a broad absorption band in the region of 3400 cm^−1^ due to the stretching vibrations of –OH and –NH_2_ groups. Features centered around 2900 cm^−1^ were present in all melanin samples and were assigned to the vibrations of the CH_2_ groups. The band at 1600 cm^−1^ was due to the C=O stretching vibration mode. The presence of the C–S stretching vibration peak at 700–600 cm^−1^ is usually used to identify the presence of pheomelanin in natural samples [22,23]. This peak was evident in the cysteinyldopa sample (Figure 3c) and was present with less intensity in the Sc-Ms1 sample (Figure 3a), suggesting the presence of pheomelanin. Furthermore, the IR profiles of these spectra were similar. The IR profile of the spectrum reported in Figure 3b (Sc-Ms1 tyrosinase dopa melanin) is similar to that reported in Figure 3d (Tv laccase dopa-melanin), with traces of a peak around 600 cm^−1^.

Natural melanin and melanin-like pigments are characterized by a persistent EPR signal due to the presence of exceptionally stable free radicals [6,29,36]. 

Normally, the melanin EPR spectra are recorded at X-band (ν ≈ 9 GHz), but at this frequency, the EPR spectrum of dopa-melanin (eumelanin) is characterized by a single slightly asymmetric line with a g-factor of ca. 2.0032 and a linewidth of 0.4–0.6 mT. This spectrum is ordinarily published to support the evident paramagnetism of the sample, but no distinctive information is usually supplied. Furthermore, the magnetic parameters are strongly dependent on pH variations and hydration conditions [8,37]. In this paper, a multifrequency EPR approach is used to describe the complex pattern of the Sc-Ms1 natural melanin based on the enzymic synthetic dopa and cysteinyldopa melanins. 

**Figure 4:**
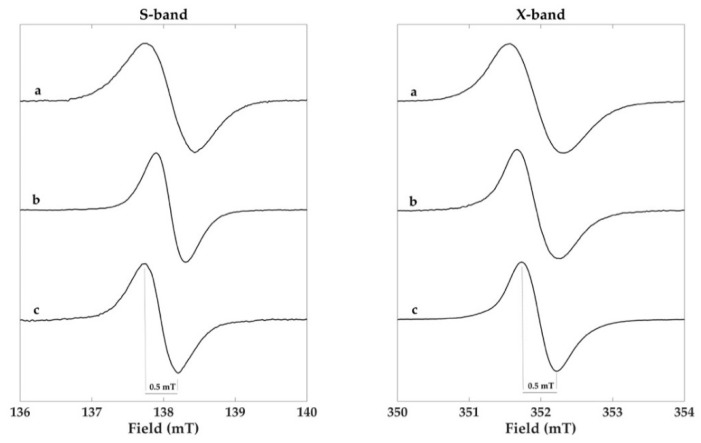
S- (3.9 GHz) and X-band (9.8 GHz) EPR spectra of the (**a**) Sc-Ms1 natural melanin, (**b**) Sc-Ms1 tyrosinase dopa-melanin, and (**c**) Tv laccase dopa-melanin samples. Spectra were recorded at 1.90 mW microwave power at S-band and 1.46 mW at X-band. Spline functions were used for the baseline correction of the S-band spectra.

In Figure 4, the X-band EPR (right side) spectrum of Sc-Ms1 melanin (Figure 4a) is compared with the spectra obtained by the reaction of Sc-Ms1 tyrosinase (Figure 4b) and Tv laccase (Figure 4c) with dopa as the substrate. All the spectra were characterized at this frequency by a single slightly asymmetric line with a linewidth of 0.7, 0.6, and 0.5 mT, respectively (Table 1). All the spectra were recorded under non-saturating conditions. 

The advantage of using a multifrequency EPR approach is that valuable informations for the sample characterization can be obtained by recording EPR spectra above and below the conventional X-band frequency (ν = 9.8 GHz). The S-band frequency (ν = 3.9 GHz) is used for a precise determination of the g isotropic value (g_iso_). At low frequencies, the spectral anisotropy is minimized, the EPR spectra are symmetric, and a direct measurement of the g_iso_ can be obtained at the crossover point on the first derivative spectrum [31]. The S-band EPR spectra for the three samples are reported in Figure 4 (left side) and the g_iso_ values in Table 1. In this context, the information derived from the analysis of the signal width (referred hereafter as peak to peak signal amplitude, ΔB_pp_) and the g values suggest that only in the case of laccase dopa-melanin the data are consistent with the formation of eumelanin (ΔB_pp_ = 0.5 mT and g = 2.0036) with the presence of carbon centered radicals. Furthermore, a certain degree of powder sample hydration cannot be excluded [8]. 

The signal amplitude and the g value of the X-band EPR spectra for the Sc-Ms1 tyrosinase dopa-melanin and Sc-Ms1 natural melanin were 0.6 and 0.7 mT and 2.0038 and 2.0047, respectively (Table 1), suggesting the presence of more than one radical species, considering that the g value for a pure eumelanin sample was reported to be 2.0032 and 2.0050–2.0055 for pure pheomelanin [8,33].

**Figure 5:**
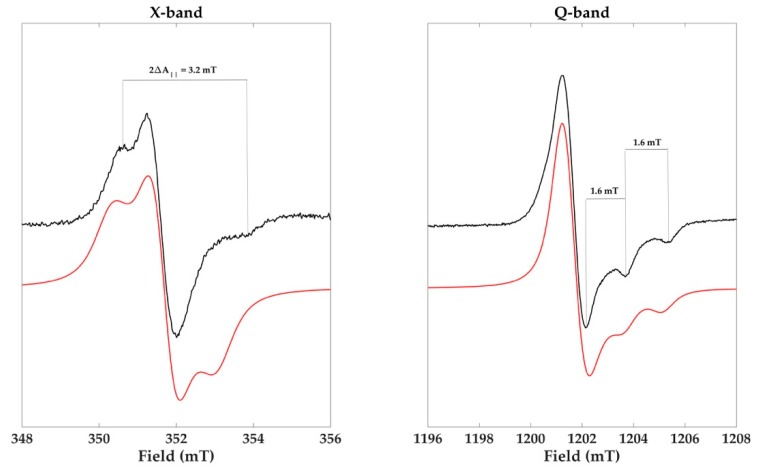
X- (9.9 GHz) and Q-band (33.7 GHz) spectra of the cysteinyldopa melanin paired with their simulated spectra (red line).

Q-band EPR experiments are crucial for addressing this point. At 35 GHz, different species can be separated based on their different anisotropies [38,39]. Given this context, cysteinyldopa melanin was synthesized using Tv laccase at neutral pH following the procedure reported in D’Ischia et al. [6] with a dopa:cysteine molar ratio of 1:2. The cysteinyldopa then polymerizes into various benzothiazine derivatives [40]. The X- and Q-band EPR spectra of the cysteinyldopa powder sample are reported in Figure 5 paired with the simulated spectra (red lines). The spectrum at X-band had a broad signal amplitude (3.2 mT) and a high g value (2.0050) and resembled the EPR spectrum of an immobilized nitroxide [6,31]. At this frequency, the EPR spectrum was dominated by the z-component of the ^14^N hyperfine splitting (Figure 5). The nitrogen coupling constant A_z_ (1.6 mT) was estimated from the Q-band cysteinyldopa spectrum, as highlighted in Figure 5. The isotropic nitrogen coupling constant (A^N^_iso_) was calculated following the same procedure as the nitroxides, assuming an axial symmetry with the tensor components of A anf g: A_x_ = A_y_ = 0.2 A_z_ and g_x_ = g_y_. The best fit, at both frequencies, was obtained using the same set of magnetic parameters reported in Table 1, changing only the frequency. The partly nitrogen-centered free radicals in cysteinyldopa melanin demonstrate the presence of the semiquinonimine radicals as was previously reported [31,41]. 

**Figure 6:**
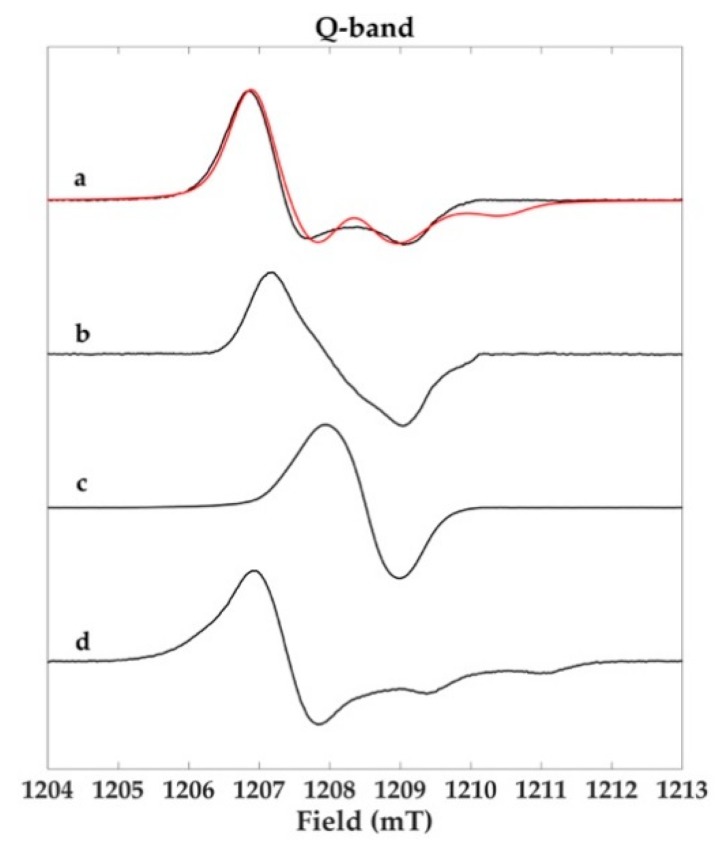
Q-band (33.9 GHz) EPR spectra of (**a**) Sc-Ms1 natural melanin, (**b**) Sc-Ms1 tyrosinase dopa-melanin, (**c**) Tv laccase dopa-melanin, and (**d**) Tv laccase cysteinyldopa melanin samples. Spectra were recorded with 0.06 mW microwave power.

In Figure 6, the Q-band spectra of Sc-Ms1 melanin and Sc-Ms1 tyrosinase melanin (Figure 6a,b, respectively) show a completely different and more complex lineshape compared to the single featureless line obtained at X-band (Figure 4a,b).

The Sc-Ms1 natural melanin showed a complex EPR signal pattern, clearly indicating the presence of more than one radical species, one of which was identified as pheomelanin contribution. Pheomelanins and eumelanins are pigments produced in humans; only few reports address the presence of pheomelanins in bacteria and fungi [21,22,23,25]. Given this context, the data reported here represent one of the first reports detecting pheomelanins in bacteria. The natural and certainly more complex melanin sample was described based on the enzymic-synthesized eumelanin and cysteinyldopa melanin (Figure 6c,d, respectively) with the Q-band spectral simulation (Figure 6a). The best fit was obtained considering the presence of the two different species and simulating the spectra with the magnetic parameters reported in Table 1. A contribution of 20% eumelanin and 80% pheomelanin was derived from the simulation. Notably, this is only a gross estimation of the two pigments’ contribution based on those synthesized by the use of Tv laccase. Furthermore, cysteinyldopa in the cells was directly synthesized through dopa cysteinylation or by the mediation of glutathione, depending on the presence of cysteine in the bacterial culture [17]. Again, the Sc-Ms1 tyrosinase was inactivated by any amount of cysteine greater than 0.01 mM [13]. The use of a cysteine-rich culture medium for Sc-Ms1 growth might justify the production of pheomelanin. The presence of the two different pigments was also confirmed by the high g value (2.0047). In the literature, a calibration curve reporting the dependence of the amount of pheomelanin on the g_exp_ factor in human red hairs was built [33]. A g value of 2.0046 was reported for a pheomelanin/eumelanin ratio of 59%, whereas a g value of 2.0038 was reported for the Sc-Ms1 tyrosinase dopa-melanin (Figure 6b), which accounts for a pheomelanin/eumelanin melanin ratio of 18%. This can be ascribed to the nature of the tyrosinase that was purified from the culture broth. Conversely, the laccase-derived sample (Figure 6c) did not show any composite signal at high frequency, supporting the assumption that laccase-derived melanin is constituted of purely heterogeneous eumelanin units. Nevertheless, the broad signal (ΔB_pp_ 1.05 mT) recorded at Q-band can account for the presence of more than one radical species. Performing hydration-controlled X-band analysis, was observed that the water content and pH can strongly influence the solid-state EPR signal [8]. A model was proposed in which two coexisting free radical species were present in an eumelanin sample, with the carbon centered (g = 2.0032) and semiquinone free radicals (g = 2.0045), whose intensity increased as pH increased. In our case, the eumelanin sample (Figure 6c) with g = 2.0036 fully agrees with the g value reported in the literature for a powder sample at neutral pH, supporting the hypothesis of carbon center free radicals with a semiquinone free radical contribution, whose formation is due to the comproportionation reaction [8]. Regardless, as Q-band frequency (ν ≅ 35 GHz) was insufficient to solve the anisotropies of these two species, higher frequencies might be desirable to determine the different contributions. The cysteinyldopa synthesis was performed using a dopa:cysteine molar ratio of 1:2. 

## 3. Materials and Methods

All chemicals and Tv laccase were obtained from Sigma Aldrich (Milano, Italy) and used without further purification. The Ms1 strain was collected and isolated from Algerian Sahara soil and was chosen for its ability to produce melanin exopigments, both in solid and liquid media. It was molecularly characterized and assigned to *S. cyaneofuscatus* Pridham et al. 1958 species [13].

### 3.1. Sc-Ms1 Tyrosinase Purification

Sc-Ms1 tyrosinase was produced by growing the strain in MPPM broth (glycerol 10 g/L, glucose 10 g/L, soya flour 10 g/L, casamino acids 5 g/L, yeast extract 5 g/L, 4.0 CaCO_3_ 4 g/L, bacteriological agar 15 g/L, and 1 mL of trace salts solution (g/100 mL: 1.0 FeSO_4_, 0.9 ZnSO_4_, 0.2 MnSO_4_), pH 7.0) supplemented with 1 mM filter sterilized CuSO_4_ for 72 h [13]. After cell harvesting by centrifuging, the tyrosinase enzyme was purified from Ms1 strain culture supernatant as previously described [13]. Briefly, proteins were precipitated from the culture supernatant with 65% ammonium sulphate, resuspended in 50 mM potassium phosphate buffer (pH 6.5), and dialyzed at 4 °C for 24 h against the same buffer. After dialysis, the enzyme solution was concentrated by replacing the dialysis buffer with a 20% (*w*/*v*) polyethylene glycol 8000 solution (in 50 mM potassium phosphate buffer, pH 6.5) and incubating at 4 °C for 24 h. The concentrated solution was finally dissolved in 50 mM potassium phosphate buffer and applied to a DEAE Sephadex ^TM^ A-50 (GE Healthcare, Waukesha, WI, USA), using batch technique to separate the enzyme from melanin.

### 3.2. Melanin Isolation and Purification 

Melanin was isolated from the Sc-Ms1 actinobacteria culture broth and purified by precipitation in an acidic environment. HCl 6 N was added to the solution until melanin precipitation. Next, the precipitate was separated from the solution by centrifugation at 15,000× *g* for 10 min at 4 °C, and washed with deionized water until the pH became neutral. 

### 3.3. Melanin-Like Pigments Synthesis by Sc-Ms1 Tyrosinase and Tv Laccase

Sc-Ms1 tyrosinase (47.6 U mg^−1^) and Tv laccase (12.9 U mg^−1^) in 100 mM phosphate buffer at pH 7 were used to synthesize eumelanin in the presence of excess dopa (6.57 mg/mL) as the substrate [6]. The reactions were followed for 16 h. The formation of an insoluble black pigment was obtained. Cysteinyldopa was synthesized using Tv laccase (12.9 U mg^−1^) with a dopa:cysteine molar ratio of 1:2. A reddish powder was obtained [6]. Samples were dried under nitrogen flux for approximately 5 h and then analyzed in the powder form.

### 3.4. UV-Visible and FT-IR Spectroscopies

A qualitative analysis of melanin samples was performed using UV-Vis near infrared (NIR) spectrometer Lambda 900/Perkin Elmer Instruments (Norwalk, CT, USA). The spectra were recorded in the wavelength range of 200 to 800 nm.

Melanin powders were IR characterized using a Nicolet FT-IR iS50 (Thermo Fischer Scientific, Madison, WI, USA). Samples were prepared as powder dispersions in KBr tablets and directly analysed.

### 3.5. Electron Paramagnetic Resonance Spectroscopy

The melanin powder samples were investigated using cw-multifrequency EPR at S-, X-, and Q-band microwave frequencies at room temperature. Each sample was prepared by transferring the melanin powder in an open EPR suprasil tubes (Cortecnet, Voisins-le-Bretonneux, France). The EPR measurements at different frequencies for each sample were collected using the same tube prepared for the Q-band measurements. All EPR microwave bridges were operating on a E580 ELEXSYS spectrometer (Bruker Biospin GmbH, Rheinstetten, Germany), equipped with the following setup: Bruker SuperQ-FT microwave bridge with *ER 5107D2* probehead for Q-band, Bruker ER 049X microwave bridge with 4122SHQE/0208 cavity for X-band, and a SB-1111 microwave bridge (Jag-Mar, Krakow, Poland) with a Loop Gap Resonator probe (Medical Advances Inc., Milwaukee, WI, USA) for S-band. All EPR spectra were recorded using the following parameters: 20 mT scan width and 0.2 mT modulation amplitude. The modulation frequency was 100 KHz for X- and S-bands and 50 KHz for Q-band measurements. A Strong Pitch standard (g = 2.0028) was used for g-value determination. Graphs were built using EasySpin package (ver. 5.2.16) on MATLAB R2017a. Simulations of the EPR spectra were performed using the routine “pepper” of the EasySpin package [42]. The automated best fittings were obtained using the Nelder-Mead (Simplex) method.

## 4. Conclusions

Melanins are heterogeneous macromolecules with persistent free radical signals. The EPR is the elective technique used for the characterization of their paramagnetism. A multifrequency EPR approach at S-, X-, and Q-bands was used to identify and characterize the melanin exopigment produced by the *Streptomyces cyaneofuscatus* actinobacteria. This brownish colored pigment revealed an EPR signal typical of melanin polymers. The strategy used here was to calculate the g_iso_ value from the S-band measurements, the linewidths and the coupling constants from the X-band spectra, and the Q-band was used to separate the different contributions based on their anisotropies. In particular, the spectral lineshape recorded at Q-band showed the presence of different contributions. Given the natural water soluble melanin complex architecture, we successfully characterized the complex in terms of eumelanin/pheomelanin enzymic synthesized pigments. The insoluble eumelanin and cysteynildopa pigments were synthesized using Tv laccase in the presence of dopa (eumelanin) and a 1:2 dopa:cysteine molar ratio (cysteinyldopa). Conversely, the Sc-Ms1 tyrosinase in the presence of dopa formed an insoluble pigment composed of an excess of eumelanin and some pheomelanin due to the natural origin of the enzyme. This study can help clarify the composition of different melanins with the valuable assistance of Q-band EPR experiments. Due to their physico-chemical characteristics, melanin pigments can be used in different applications, ranging from UV-Vis protectants, radical scavengers, conductive materials, electrochemical applications, and in medical applications to understand melanin related diseases. In this context, for a better understanding of the electronic structure of different melanins and their characterization in solid states, microwave power saturation EPR measurements and pulsed Q-band relaxation experiments are being undertaken.

## Figures and Tables

**Table 1:** Magnetic parameters for the natural and enzymic synthetic melanin samples.

Sample	A^N^_iso_	A^N^_z_	2A^N^_z_	g_iso_ *	g_z_	g_x_ = g_y_	ΔB_pp_^§^ (mT)
Sc-Ms1 melanin				2.0047			0.7
Sc-Ms1 Tyr. dopa melanin				2.0038			0.6
Tv Lac. dopa melanin				2.0036			0.5
Tv Lac. cysteinyldopa melanin	0.7	1.6	3.2	2.0050	2.0028	2.0060	3.2

* determined from the S-band (3.8 GHz) electron paramagnetic resonance (EPR) spectra; ^§^ determined from the X-band (9.8 GHz) EPR spectra. Errors were estimated to g values ±0.0002 and hyperfine splittings ±0.05 mT.
